# The combined effect of family environment and parents' characteristics on the use of food to soothe children

**DOI:** 10.1002/fsn3.3941

**Published:** 2024-01-31

**Authors:** Mar Lozano‐Casanova, Isabel Sospedra, Antonio Oliver‐Roig, Miguel Richart‐Martinez, Ana Gutierrez‐Hervas

**Affiliations:** ^1^ Nursing Department, Faculty of Health Science University of Alicante Alicante Spain

**Keywords:** calm, child nutrition, child obesity, family relations, feeding behavior, feeding practices, parenting

## Abstract

Parental feeding practices, such as the use of food to soothe, can be shaped by various factors, including the family environment and parents' psychological characteristics and capacities. To our knowledge, the combined effect of these factors has not been studied. Furthermore, parental feeding practices have mainly been studied in women, resulting in a gender gap in the research. This study aims to investigate the combined effect of family environment and parental characteristics on the likelihood of using food to soothe children, taking the gender of both parents into account. This cross‐sectional study included a sample of 846 parents (36.3% men) of 1‐year‐old children from different regions of Spain. Participants completed an online survey that included questionnaires to measure whether parents used food to soothe children, the family environment, parents' characteristics, and their psychological capacities. Binary logistic regression analyses were performed to identify associations between the variables. The final model showed that, within the family environment, higher levels of dyadic adjustment between couples (OR = 0.965; *p* = .026) were associated with a reduced likelihood of using food to soothe children, whereas the psychological characteristic of parental fatigue (OR = 1.053; *p* = .007) appeared to be associated with an increased likelihood. Also associated with an increased likelihood of this practice were higher parental sense of competence (OR = 1.028; *p* = .029) and the attention dimension of emotional intelligence (OR = 1.043; *p* = .007). Our study suggests that using food to soothe children may be influenced by factors at different levels, from the quality and adjustment of the couple's relationship to parental fatigue, self‐competence, and emotional intelligence. For future research, it may be worthwhile contextualizing parental practices to gain a better understanding of children's behavior.

## Graphical abstract

Our results suggested that having good quality and adjustment in the relationship between partners reduced the risk of using food to calm children, whereas the highest levels of fatigue, parent sense of competence, and attention to the emotions increased it. A positive family environment, parents' mental well‐being, and self‐capacities have an effect on parents' feeding practices on their children. Expanding our knowledge about which factors could be associated with different feeding practices is relevant to prevent children obesity and other non‐communicable diseases.

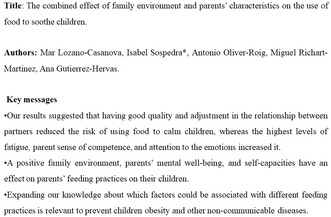

## 1 INTRODUCTION

The use of food to soothe infants is a very common feeding practice among caregivers. It is traditionally used to deal with crying babies, as crying is seen as a sign of hunger (Harrison et al., 2017). However, many parents also view this practice as an effective way of soothing their children even when they are not hungry (Orr et al., 2019). Recent literature suggests that this and other feeding practices may have a negative impact on the risk of obesity and emotional eating by encouraging children to use food for emotional regulation and comfort (Adams et al., 2019; Freitas et al., 2018; Hampl et al., 2023; Jansen et al., 2019; Savage et al., 2018; Shloim et al., 2015; Stifter et al., 2011). Indeed, childhood overweight and obesity are now recognized as global public health problems due to their high prevalence and adverse health implications later in life (Hampl et al., 2023).

When examining the practice of using food to calm children from a socioecological perspective, it is important to consider not only the type of food provided but also the factors that lead parents to use food rather than other more appropriate strategies or tools (Moore et al., 2013).

There are three interrelated levels at which human behavior, including parental feeding practices, may be influenced (Bronfenbrenner, 1995). First, there is the social level, which includes cultural norms, societal gender roles, and economic policies (Hector et al., 2005). This implies differences in parental feeding practices across cultures and ethnic groups (Briefel et al., 2010; Cardel et al., 2012; Haines et al., 2019; Huang et al., 2012).

At a closer level, more specifically within the family context, positive environments (e.g., less conflict and more positive emotions at mealtime) are associated with better child behavior and more responsive parenting and feeding practices (Chan et al., 2018; Scaglioni et al., 2018; Wolstenholme et al., 2020) and thus better outcomes for children (Adams et al., 2019; Wolstenholme et al., 2020). A functional and cooperative relationship between parents, in particular, reduces instrumental and nonresponsive feeding practices, such as the use of food to soothe children (Sleddens et al., 2014; Thullen et al., 2016).

Finally, at the individual level, characteristics such as maternal stress and anxiety, as well as lower parenting satisfaction, are positively associated with greater use of unresponsive practices and uninvolved or controlling feeding styles (Doan et al., 2022; El‐Behadli et al., 2015; Hughes et al., 2015). Furthermore, the symptoms of anxiety, fatigue and insomnia, and negative emotions in general are related to increased emotional eating in adults, which in turn leads to a tendency to do the same with children (Rodgers et al., 2014; Yoshikawa et al., 2014; Zhu et al., 2019).

In addition to these psychological characteristics, research at an individual level has also looked at psychological capacities such as self‐efficacy and parental competence. Parental self‐efficacy appears to lead parents to adopt child feeding practices in line with infant feeding recommendations (more responsive feeding strategies and increased consumption of fruit and vegetables) (Bahorski et al., 2019; Möhler et al., 2020). Furthermore, a lower parental sense of competence correlates to more controlling behavior and problems during family mealtimes (Aviram et al., 2015).

To our knowledge, the effects of family environment (Scaglioni et al., 2018; Thullen et al., 2016), individual characteristics (Doan et al., 2022; El‐Behadli et al., 2015; Hughes et al., 2015; Zhu et al., 2019), and psychological capacities (Aviram et al., 2015; Bahorski et al., 2019; Möhler et al., 2020) have only ever been studied separately, and there is a lack of evidence on their combined effect on feeding practices. Furthermore, parental feeding practices and styles have mainly been studied in women, without including men in the sample (Pratt et al., 2019; Tschann et al., 2013). There is a gender gap in the current literature that needs to be evaluated to determine whether there are gender differences in parenting and feeding practices, as suggested by some research (Pratt et al., 2019).

This study, therefore, aims to explore the combined effect of family environment together with parental characteristics on the likelihood of using food to soothe children, taking the gender of both parents into account. Our hypothesis is that this practice of soothing children will be influenced by both levels (family and individual characteristics) and that differences will be found according to the parents' gender.

## 2 METHODS

### 2.1 Design

This cross‐sectional study was part of wider research into positive parenting practices that were carried out with a 3‐year follow‐up. It involved a convenience sample of parents with children aged 12–24 months in eastern Spain.

The sample included women and their partners with sufficient literacy in Spanish and access to internet‐enabled electronic devices. The participants had also fully completed the questionnaires of interest to us in this study. Parents of children with serious medical conditions, children with pathologies with nutritional implications, or children with developmental abnormalities at birth were excluded.

### 2.2 Ethics

The study was conducted in accordance with the Declaration of Helsinki. Ethical approval was obtained from the Clinical Research Ethics Committee of the General Directorate of Public Health and the Higher Public Health Research Centre of the Valencia Ministry of Health.

Participants were informed of the objective of the study and given a signed written informed consent form.

### 2.3 Data collection

Initial contact was made during the postpartum hospital stay, when information about the study was provided in writing and consent was obtained. Participants were asked to complete a self‐administered questionnaire to collect their sociodemographic characteristics and contact information for follow‐up. Participants received an email informing them that they would receive further questionnaires at different points in time. The first postpartum follow‐up was sent to gather information about the baby's first months of life. The second self‐reported online survey, which included the various questionnaires used in this study, was then sent between 12 and 24 months postpartum. The online questionnaire could be completed over several sessions. The responses of each participant were given an identification code. During the following 3 weeks, email and SMS reminders were sent to mothers who had not responded to the survey, and a prize draw of 300 € was held among study participants as a way of boosting the response rate.

### 2.4 Variables

#### 2.4.1 Outcome variable: Caregivers' use of food to soothe children

We used a specific item, based on one item included in the BBNQ questionnaire (Stifter et al., 2011), relating to the use of food to soothe children: “How often do you offer food to calm your child when he/she cries or is fussy?” The item was scored on a 5‐point Likert scale from 1 (never) to 5 (always), where the sum of “sometimes,” “almost always,” and “always” was used to indicate the use of food to soothe children, and “rarely” and “never” indicated that it was not used.

#### 2.4.2 Predictive variables

*Family context*

*Social support from COOP/WONCA charts (COOP/WONCA)*. COOP/WONCA is used to measure health‐related quality of life (HRQoL). It is a nine‐item questionnaire in which each dimension has its own heading and asks a question related to events in the previous month. The chart headings are physical fitness, feelings, daily activities, social activities, changes in health, health condition, pain, social support, and quality of life. Higher scores correspond to poorer HRQoL (Lizán Tudela & Reig Ferrer, 2002).

*Dyadic adjustment scale (DAS)*. The dyadic adjustment scale assesses the quality and adjustment of a couple's relationship. It consists of 13 items with three interrelated subscale (satisfaction, consensus, and cohesion) scores and an overall score. Higher scores indicate better relationship adjustment (Santos‐Iglesias et al., 2009).

*Parental alliance inventory (PAI)*. The PAI is a 20‐item instrument measuring parental alliance in relation to the following areas: each parent's investment in the child, how each parent rates the other's involvement with the child, each parent's respect for the other's judgments, and each parent's desire to communicate with the other (Menéndez et al., 2012).

2.4.3

*Individual characteristics*

*Warwick–Edinburgh mental well‐being scale (WEMWBS)*. This scale covers both hedonic and eudaimonic aspects of mental health, including positive affect, satisfying interpersonal relationships and positive functioning. The WEMWBS is a 14‐item instrument using a 5‐point Likert scale from 1 (none of the time) to 5 (all of the time) to describe the individual's experience of each statement over the previous 2 weeks. The minimum score is 14, and the maximum is 70 (all scores are positive). Higher scores indicate greater mental well‐being (López et al., 2013).

*Parental stress scale (PSS)*. The PSS is a 17‐item scale measuring the parental stress experienced as a result of parenting using a 5‐point Likert scale ranging from 1 (strongly disagree) to 5 (strongly agree) (Oronoz et al., 2007).

*Fatigue assessment scale (FAS)*. This 10‐item scale measures fatigue levels with five items assessing the physical dimension and five assessing the psychological dimension, yielding a single score from 10 to 50. It is a 5‐point scale ranging from 1 (never) to 5 (always) (Cano‐Climent et al., 2017).

*Athens insomnia scale (AIS)*. The Athens Insomnia Scale assesses insomnia during the month prior to completion of the questionnaire. It consists of eight items: 1–4, evaluate sleep duration; 5, sleep quality; and 6–8, how insomnia affects the person during the day. It is a scale with five possible scores from 1 to 5, where 5 represents the highest level of difficulty (Portocarrero & Jiménez‐Genchi, 2005).

*Dimensions of caregivers' feeding styles*. Caregiver responsiveness (how much the parent encourages eating) and demandingness (whether the parents encourage eating in a responsive or nonresponsive way) (Hughes et al., 2005) levels were established using the Spanish version of the Toddler Feeding Style Questionnaire (TFSQ) (Avecilla‐Benítez et al., 2019). This assesses the parental feeding styles in children between 12 and 24 months old. The TFSQ contains six items measuring each dimension (six for demandingness and six for responsiveness).

2.4.4

*Psychological capacities*

*Parental sense of competence scale (PSOC)*. This instrument is a 17‐item scale measuring perceived parental competence on a 6‐point Likert‐type scale from 1 (strongly disagree) to 6 (strongly agree). A higher score indicates higher parental sense of competence (Oltra‐Benavent et al., 2020).

2.4.5

*Trait Meta‐Mood scale (TMMS‐24)*

*The TMMS‐24 assesses each person's perception of their attention (the degree to which people name and think about their emotions), clarity (the degree to which people clearly perceive their emotions), and repair (the degree to which people regulate their emotions)* (Berrocal & Diaz, 1999). The following sociodemographic variables were analyzed for the caregivers: age, gender, nationality (Spanish/non‐Spanish), level of education (secondary school completed/not completed), socioeconomic status in terms of annual income and employment status (unemployed/employed). The following variables were dichotomized: nationality (Spanish or other); annual income dichotomized to reflect the Spanish minimum wage of approximately 1000 Euros/month (Ministerio de Trabajo y Economía Social, 2022) (<12,000 Euros or >12,000 Euros per annum); previous parenting experience, given the challenges involved in the transition to parenthood (Lévesque et al., 2020) (first child or more); employment status (employed or unemployed); educational attainment (up to secondary or undergraduate level); and the use of food to soothe children where the sum of “sometimes,” “almost always,” and “always” was used to indicate the use of food to soothe children, and “rarely” and “never” to indicate that it was not used.

### 2.5 Statistical analyses

Descriptive analyses of all variables included percentages and the mean was calculated for obtaining parents' age and the median for the scores of the predictable variables as they are continuous variables. The standard deviation was calculated in both cases.

Initially, univariate logistic regressions were conducted to establish crude odds ratio values using food to soothe children as the outcome. Subsequently, employing the conditional method, multiple binary logistic regressions were performed to refine associations between variables and derive a comprehensive final model incorporating all factors. No imputations for missing data were made, and SPSSv23.0 statistical software was used to perform all the analyses.

## 3 RESULTS

### 3.1 Sample characteristics

Table 1 shows the sample's sociodemographic characteristics. A total of 1205 participants were recruited in the study, approximately 40% of whom were men. The study participants (Figure S1) had a mean ± SE age of 34.71 ± 4.22 years old. The vast majority of participants were Spaniards with a high school education, actively employed, and with an annual income of more than 12,000 euros.

**TABLE 1:** Participants' sociodemographic characteristics including gender, age, nationality, income, work and educational status, and parenting experience.

	*n*	%
Gender	846	
Men	307	36.3
Women	539	63.7
Nationality	840	
Spanish	799	95.1
Other	41	4.9
Annual salary (€/year)	789	
<12,000	126	16
>12,000	663	84
Previous parenting experience	846	
First child	502	59.3
Previous children	344	40.7
Employment status	471	
Employed	364	77.3
Unemployed	107	22.7
Education level	817	
Secondary school	216	26.4
Higher education (undergraduate)	601	73.6

Table 2 shows the descriptive results for the different variables included in the logistic regressions to obtain the final model for parental use of food to soothe children. Of the total sample, 846 participants responded to the BBNQ item on the use of food to soothe children, with only 25% tending to do so. The response rate in variables related to the family context was the lowest level (62.65%) in comparison with the more than 90% response rate in the individual characteristics and psychological capacities variables.

**TABLE 2:** Baseline sample characteristics for child feeding, psychological, couple's relationship, and social support variables.

	*n*	%	Mdn ± DS
Using food to soothe children	846	100	
Yes	211	24.9	
No	635	75.1	
Demandingness	841	99.41	28 ± 7.85
Responsiveness	842	99.53	33 ± 5.1
FAS	812	95.98	19.5 ± 7.23
Social support COOP/WONCA charts	753	89.01	2 ± 1.05
AIS	804	95.04	5 ± 4.12
WEMWBS	795	93.97	55 ± 8.35
PSS	846	100	24 ± 6.68
DAS	530	62.65	50 ± 7.55
PSOC	845	99.88	77 ± 11.62
PAI	530	62.65	90 ± 9.87
Attention TMMS‐24	772	91.25	23 ± 6.29
Clarity TMMS‐24	772	91.25	28 ± 6.61
Repair TMMS‐24	772	91.25	28 ± 6.55

Abbreviations: AIS, Athens Insomnia Scale; DAS, Dyadic Adjustment Scale; FAS, Fatigue Assessment Scale; Mdn, median; PAI, parental alliance inventory; PSOC, Parental Sense of Competence Scale; PSS, Parental Stress Scale; SD, standard deviation; TMMS‐24, Trait Meta‐Mood Scale; WEMWBS, Warwick–Edinburgh Mental Well‐Being Scale.

### 3.2 Factors influencing the use of food to calm children

The results obtained from the binary logistic regressions are shown in Table 3.

**TABLE 3:** Caregiver predictors of the use of food to soothe children.

	Use of food to soothe children (*n* = 398)			
Predictor variable	Crude regression, OR (95% CI)	*p*‐value	Binary logistic regression, OR (95% CI)	*p*‐value
Spanish nationality	0.899 (0.442–1.826)	.767		n.s.
First child	0.748 (0.546–1.024)	.070		n.s.
Gender	1.199 (0.864–1.666)	.278		n.s.
Education level	0.759 (0.522–1.102)	.759		n.s.
Household income	0.888 (0.566–1.393)	.605		n.s.
Age	1.003 (0.966–1.042)	.876		n.s.
Employed	0.800 (0.563–1.138)	.214		n.s.
Social support COOP/WONCA	1.095 (0936–1.282)	.257		n.s.
Responsiveness	1.023 (0.992–1.056)	.149		n.s.
Demandingness	0.995 (0.976–1.015)	.629		n.s.
PAI	0.988 (0.969–1.007)	.208		n.s.
WEMWBS	0.978 (0.960–0.997)	.023*		n.s.
AIS	1.054 (1.015–1.094)	.006**		n.s.
PSS	1.036 (1.013–1.060)	.002**		n.s.
Clarity TMMS‐24	0.996 (0.972–1.021)	.758		n.s.
Repair TMMS‐24	0.999 (0.974–1.024)	.929		n.s.
Attention TMMS‐24	1.044 (1.017–1.072)	.001**	1.053 (1.015–1.094)	.007**
FAS	1.034 (1.012–1.056)	.002**	1.053 (1.014–1.094)	.007**
PSOC	0.991 (0.978–1.004)	.164	1.028 (1.003–1.053)	.029*
DAS	0.960 (0.936–0.984)	.001**	0.965 (0.935–0.996)	.026*

*Note*: Multinomial logistic regression R^2^ Cox–Snell = 0.066, R^2^ Nagelkerke = 0.097. Model χ2 (17) = 27.022, *p* < .001. Odds ratios (OR) from logistic regression models included all sociodemographic characteristics, parental well‐being variables, and feeding styles dimensions.

* *p* < .05.

** *p* < .01.

Abbreviations: AIS, Athens Insomnia Scale; DAS, Dyadic Adjustment Scale; FAS, Fatigue Assessment Scale; n.s., nonsignificant; PAI, parental alliance inventory; PSOC, Parental Sense of Competence Scale; PSS, Parental Stress Scale; TMMS‐24, Trait Meta‐Mood Scale; WEMWBS, Warwick–Edinburgh Mental Well‐Being Scale.

Following binary logistic regression, the accuracy of the adjusted model was found to be 74.9%. Higher levels of fatigue, the attention dimension of the TMMS‐24, and the parental sense of competence (PSOC) were associated with using food to soothe children with statistical significance. The family environment variable that was statistically significant in reducing the use of this practice was DAS.

## 4 DISCUSSION

The principal aim of this study was to explore the combined effect of family environment and individual characteristics and psychological capacities on parental feeding practices. Overall, the results in our final model revealed that when all variables were combined, the different levels studied (family environment, individual characteristics, and psychological capacities) had an effect on parental use of food to soothe children. In this respect, good quality and adjustment in a couple's relationship (positive family environment) were associated with a lower likelihood of using food to soothe children. Meanwhile, higher levels of fatigue (individual characteristics), parental sense of competence, and attention to emotions (psychological capacities) were associated with an increased likelihood of using this feeding practice. Although some literature suggests differences in feeding styles and practices between genders (Barrett et al., 2018; Pratt et al., 2019; Tschann et al., 2013), our findings showed no statistical significance for the gender variable alone, so it was not included in the final model. By examining each variable's individual contribution, we found that mental well‐being could be associated with a reduced likelihood of using food and higher levels of insomnia and parental stress with an increased likelihood. However, these individual effects were lost once all variables were combined.

With regard to the family environment, the association found between good quality and adjustment of the couple's relationship and a lower likelihood of using food to soothe children is supported by the literature. This research links better feeding practices and eating behaviors with healthy family environments and dynamics. It is suggested that this may be due to the effect of role modeling, or support for the adoption of healthy eating behaviors learned through observation (Scaglioni et al., 2018; Sleddens et al., 2014).

Several researchers have examined the association between higher levels of various parental distress symptoms and the use of food for non‐nutritional purposes or other less responsive practices (Doan et al., 2022; Jang et al., 2019; Zysberg, 2018). In our final model, we found that fatigue was the parental distress symptom that most increased the likelihood of using food to calm children. In fact, fatigue is associated with reduced cognitive and physical functioning, increased parental stress, and irritability during interactions with children, all of which contribute to poor parenting practices (Martins, 2019; Wilson et al., 2019). Moreover, our results showed that other symptoms of distress, such as insomnia and parental stress, also appeared to increase the likelihood of using food to calm children when binary regressions were performed. All of these symptoms can lead parents to resort to less effortful ways of coping with their children's cries. In fact, some parents agreed that feeding is the best way to stop a baby crying (Orr et al., 2019).

Traditionally, a baby's cry has been seen as a sign of hunger, and the first response is to use food to calm them (Harrison et al., 2017; Hidalgo García & Menéndez Álvarez‐Dardet, 2009). Furthermore, the act of providing food has been used to show affection and support to loved ones (Hamburg et al., 2014). In both cases, if parents see the baby's crying as a sign of hunger or if they reach for food because they are worried about their child's discomfort and want to make them feel better and supported, giving food in response may increase their perceived confidence in their parenting role because they believe they are responding to their child's needs. This could explain why parents with the highest scores for PSOC were those who used food to soothe their child, in the belief that they were responding to their child's discomfort.

These results regarding the PSOC may also be related to the presence of the attention dimension of the TMMS‐24 (emotional intelligence) in our final model, which increases the likelihood of using food to calm children. In this sense, emotional intelligence seems to be associated with more effective skills in managing emotions and recognizing them (Zysberg, 2018). Thus, parents with higher emotional intelligence or those who feel more competent may have high levels of responsiveness and perhaps also high levels of demandingness. Nevertheless, this is not linked to better compliance with nutritional recommendations for children. It is also interesting to note the evidence suggesting that excessive emotional awareness can also be associated with the increased use of food for emotional management and eating disorders (Braden et al., 2018; Sanchez‐Ruiz et al., 2019), rather than for nutritional purposes. In this regard, parents who use food for comfort may take the same approach with their children, that is, by using food to soothe them.

However, at this point, it is important to consider the reasons for the child's behavior (infant crying or other disturbed behavior) to classify the practice of using food to soothe infants as either nonreactive or reactive. As previously suggested, these may be due to physiological needs such as hunger (Harrison et al., 2017; Hidalgo García & Menéndez Álvarez‐Dardet, 2009) but may also be due to the family environment. There is evidence of an interaction between children's temperament and the family environment (Hudson et al., 2011). For example, it has been shown that in families with negative environments (e.g., high levels of conflict), children are more prone to develop dysregulated or maladaptive responses to stress (Lucas‐Thompson & Goldberg, 2011). In these terms, we should therefore consider that parents with high self‐competence may be better able to detect when their babies cry out of hunger or discomfort and respond to this by using food in a responsive way. In addition, as children in households with a poor quality family environment display more maladaptive behaviors, parents, out of desperation or exhaustion, may decide to use food to soothe the children as an easy and effective way to cope (Orr et al., 2019). In this case, it is considered a nonresponse mechanism. Conversely, as our results suggest, good quality and adjustment in the relationships between family members (positive family environment) can help to improve children's behaviors (Hudson et al., 2011), and as a consequence, parents would not have the necessity of resorting to food to manage their children's behaviors.

One of the main limitations of this work is that it failed to take into account the reason for children's disturbed behavior and the type of food provided to calm them down. Consequently, the use of food to soothe children cannot be classified as either a responsive or nonresponsive feeding practice. As such, we believe that future research in this field should consider the underlying reasons for children's behavior. Second, the study was mainly carried out in a population from eastern Spain, and extrapolating its results to other cultures or contexts would not be advisable. A further limitation is that our participants were recruited using an accidental sampling method. As a result, although our sample included men, by virtue of the fact that they chose to participate in this study, their attitudes and behaviors in relation to parenting may be different from those of other fathers who did not take part. Finally, due to the cross‐sectional nature of the data analysis procedures, a causal relationship cannot be established.

To our knowledge, this study is the first of its kind to provide information on the combined effect of different variables measuring the family environment and parents' individual characteristics and psychological capacities on the use of food to calm children. Although using food to cope with crying children is a widespread approach passed on from one generation to the next (Harrison et al., 2017), we should stress that it is not recommended when used for non‐nutritional purposes (Savage et al., 2018). In such cases, it should not be encouraged. We would like to highlight the importance of family support and personal resources in helping people cope with their symptoms of distress, which seem to have a major impact on parental attitudes and behaviors, particularly in this study on the use of food to soothe children. For this reason, it may be worthwhile to develop training sessions to teach parents more appropriate strategies for managing their children's negative emotions, such as swaddling or rocking them (Savage et al., 2018).

## 5 CONCLUSIONS

In summary, the results of this study suggest that a positive family environment and individual characteristics and psychological capacities influence feeding practices. Our findings on parental competence and emotional intelligence show the influence of personal beliefs and social context on parental feeding practices. This highlights the importance of taking into account parents' beliefs and interpretations of their children's behavior when studying feeding practices.

Further research is recommended to identify which factors could be associated with noninstrumental feeding practices. This information will enable professionals to provide parents with the tools and strategies they need to cope with children's crying and identify when this is a sign of hunger. The result could be an improvement in the physical and mental health of not only the child but also the whole family.

## AUTHOR CONTRIBUTIONS

Mar Lozano‐Casanova, Ana Gutierrez‐Hervas, and Isabel Sospedra performed the research; Miguel Richart‐Martinez, Antonio Oliver‐Roig, and Isabel Sospedra designed the research study; Ana Gutierrez‐Hervas, Isabel Sospedra, and Miguel Richart‐Martinez contributed essential reagents and tools; Mar Lozano‐Casanova and Antonio Oliver‐Roig analyzed the data; Mar Lozano‐Casanova and Isabel Sospedra wrote the first draft of the paper; Ana Gutierrez‐Hervas, Antonio Oliver‐Roig, and Miguel Richart‐Martinez provided critical input to the manuscript. All authors approved the final version of the manuscript.

## FUNDING INFORMATION

Mar Lozano‐Casanova would like to thank the Ministerio de Universidades for the FPU21/04232 grant.

## CONFLICT OF INTEREST STATEMENT

The authors declare that they have no competing interests.

## ETHICS STATEMENT

Ethical approval was obtained from the Clinical Research Ethics Committee of the General Directorate of Public Health and the Higher Public Health Research Centre of the Valencia Ministry of Health.

## Supporting information


Figure S1.

## Data Availability

The data that support the findings of this study are available upon request from the corresponding author.
