# Correction to “The Combined Effect of Family Environment and Parents’ Characteristics on the Use of Food to Soothe Children”

**DOI:** 10.1002/fsn3.71305

**Published:** 2025-11-28

Lozano‐Casanova M, Sospedra I, Oliver‐Roig A, Richart‐Martinez M, Gutierrez‐Hervas A. The Combined Effect of Family Environment and Parents’ Characteristics on the Use of Food to Soothe Children. *Food Sci Nutr*. 2024;12:2588–2596. 10.1002/fsn3.3941.

In the original publication of the article, the following funding information was missing:

This research was also supported by the emerging project GRE21‐08 from the University of Alicante.

We apologize for this error.

