# Supplementary material for: The combined effect of family environment and parents' characteristics on the use of food to soothe children
Source: Food Sci Nutr. 2024 Jan 31;12(4):2588–96. doi: 10.1002/fsn3.3941 (PMC11016393; doi:10.1002/fsn3.3941)
Supplement: Supplementary file 1 — Figure S1. [file FSN3-12-2588-s001.docx]

Initial sample included in the large study

n= 3905

Missed for no having email

n= 823

Missed for incomplete questionnaire at the beginning of the longitudinal study

n= 128

Missed from one to six months follow-up

n=1610

n= 1610

Missed at 12 months follow-up

n= 139

Sample included in the present study

n= 1205

Supplementary Figure 1. Participant flow chart showing how the sample of this study was obtained.
